# Enhancing production and assessing IgE reactivity of dog allergen Can f 6 in *Pichia pastoris* and *Escherichia coli*

**DOI:** 10.1007/s00253-025-13465-7

**Published:** 2025-03-29

**Authors:** Juta Dvareckienė, Gintautas Žvirblis, Mindaugas Zaveckas, Rasa Petraitytė-Burneikienė

**Affiliations:** Vilnius University, Life Sciences Center, Institute of Biotechnology, Sauletekio Av. 7, 10257 Vilnius, Lithuania

**Keywords:** Can f 6, Glycoprotein, Recombinant, *Pichia pastoris*, ELISA, MBP

## Abstract

Pet allergies are increasingly prevalent in developed nations, significantly affecting humans and strongly linked with asthma and rhinitis. Allergic reactions to cats and dogs affect 15.7% of Americans and 27.2% of Europeans, with sensitization rates to dog allergens reaching 56.0% in Denmark. Despite these concerns, dog ownership remains widespread, with 25% of European and 45.5% of US households owning at least one dog. With sensitization on the rise and current diagnostic and therapeutic approaches predominantly relying on inherently inconsistent allergen extracts derived from natural sources, recombinant allergen production offers a pathway to component-resolved diagnostics, improving specificity and reliability in allergy diagnosis. The present research explored, for the first time, the production of the allergen component glycoprotein Can f 6 in the eukaryotic expression system *Pichia pastoris* and compared its IgE antigenicity to recombinant Can f 6 (rCan f 6) variants produced in *Escherichia coli*. Yields were significantly increased by fusing Can f 6 with the maltose binding protein (MBP), resulting in a 1.8-fold increase in production in *E. coli* and a threefold increase in *P. pastoris*. Antigenicity analysis showed that N-glycosylation is not critical for folding or IgE recognition of Can f 6, making both systems equally suitable for producing the allergen. Notably, *P. pastoris*-produced MBP fused protein purified through cation exchange chromatography yielded a lower protein quantity. Still, it exhibited stronger IgE reactivity than the same protein purified using anion exchange chromatography.

**Graphical abstract:**

**Figure:**
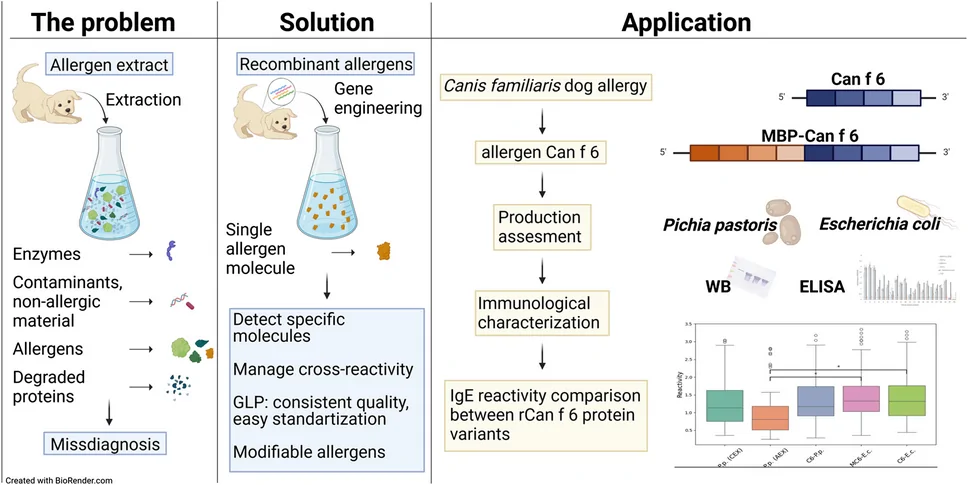

**Supplementary Information:**

The online version contains supplementary material available at 10.1007/s00253-025-13465-7.

## Introduction

Research indicates an increasing prevalence of pet allergies in developed nations, emphasizing the concerning association between these allergies and the development of asthma and rhinitis (Konradsen et al. 2015). In the USA, up to 15.7% of those aged six years and older report experiencing allergic reactions to cats and dogs (Salo et al. 2014). Similarly, Europe demonstrates a comparable pattern, with dog allergen sensitization averaging 27.2% and reaching as high as 56.0% in Denmark (Heinzerling et al. 2009). Despite these concerns, dog ownership remains widespread, with recent surveys indicating that approximately 25% of households in Europe and nearly 45.5% in the USA own at least one dog (European Pet Food Industry Federation (FEDIAF) 2024; American Veterinary Medical Association (AVMA) 2024).

The domestic dog, *Canis familiaris*, is a significant source of indoor allergens, with allergens sequentially designated from Can f 1 to Can f 8 (www.allergen.org n.d). These allergens are found in various biological materials, including dander, sweat, saliva, and urine. Among them, Can f 1, Can f 2, Can f 4, and Can f 6 belong to the lipocalin protein family, a major group of mammalian aeroallergens ubiquitously present in animals, plants, and bacteria (Konieczny et al. 1997; Kamata et al. 2007; Mattsson et al. 2010; Nilsson et al. 2012; Hilger et al. 2012). In contrast, Can f 3, Can f 5, Can f 7, and Can f 8 have been identified as serum albumin, prostatic kallikrein, Niemann pick type C2 protein, and cystatin A, respectively (Pandjaitan et al. 2000; Mattsson et al. 2009; Khurana et al. 2016; Roesner et al. 2022).

In allergology, particularly regarding dog allergies, lipocalin family members are significant as they represent cross-reactive molecules that could facilitate allergic reactions across various species (Hilger et al. 2012). Lipocalins are small extracellular molecules characterized by a common tertiary structure composed of eight anti-parallel β-strands forming a β-barrel structure with one α-helix (Yamamoto et al. 2019). Previous observations show that within the β-barrel is a hydrophobic cavity able to bind small hydrophobic molecules such as retinol, steroids, odorants, and pheromones. These small molecules are secreted into the environment, gradually releasing odorants and extending their longevity (Tegoni et al. 2000; Hilger et al. 2012). The prolonged environmental persistence of these molecules enhances their allergenic potential, as extended-release increases the exposure period for sensitized individuals.

Can f 6 is the most recently characterized lipocalin allergen, identified in dog saliva and dander (Nilsson et al. 2012; Polovic et al. 2013). Its crystalline structure classifies it as a polyvalent allergen with highly structure-dependent epitopes, displaying significant variations in IgE reactivity between its structured and denatured forms (Yamamoto et al. 2019). Conformational epitopes, rather than primary sequence identity, are recognized as the leading cause of cross-reactivity among allergens (Jimenez-Lopez et al. 2012). Specific IgE (sIgE) to Can f 6 appear in 38–56% of dog-sensitized subjects; however, that figure can rise to 61% when considering cross-reactivities with other lipocalin allergens like the cat allergen Fel d 4 and horse allergen Equ c 1 (Nilsson et al. 2012; Hilger et al. 2012; Wang et al. 2017; Yamamoto et al. 2019). These findings underscore the complex role of Can f 6 in pet allergies and its relevance in cross-species allergenic responses.

Although the structural significance of lipocalin allergens like Can f 6 is well-established, until now, it has only been synthesized in *Escherichia coli* (Nilsson et al. 2012). Despite its diagnostic potential, recombinant Can f 6 (rCan f 6) is not universally used for dog allergy diagnosis as allergen extracts from natural sources—with their inherent limitations—remain the primary basis for allergy diagnosis and allergen immunotherapy (AIT) (Valenta et al. 2018). Allergen component rCan f 6 can be found in ImmunoCAP® (Phadia, Thermo Fisher Scientific) singleplex and ALEX® (MacroArray Diagnostics) multiplex assays; however, the manufacturers behind these assays did not disclose the full details of their production, so the expression system used to produce rCan f 6 cannot be determined. In contrast, other systems—such as ImmunoCAP® ISAC (Phadia, Thermo Fisher Scientific), Immulite (Siemens Healthineers), NOVEOS (HYCOR Biomedical), and various indirect enzyme-linked immunosorbent assays or Lateral Flow immunoassays—do not include Can f 6 in their analysis. Studies comparing allergen components with extracts for predicting dog allergy severity consistently demonstrate that measuring sIgE using components provides greater diagnostic accuracy (Schoos et al. 2021). Substituting traditional allergen extracts with recombinant or synthetic ones thus represents advancement toward component-resolved diagnostics (CRD) (Curin et al. 2017).

The objective of this study was to evaluate the production of the glycoprotein Can f 6 in a eukaryotic expression system, focusing on its ability to specifically recognize sIgE antibodies, its non-reactivity toward non-specific IgE, and the feasibility of high-yield production for large-scale applications. Anticipating potential pharmaceutical applications of rCan f 6, we explored methodologies to augment protein yield and improve sIgE recognition. The methodological approach taken in this study included fusing Can f 6 with the maltose binding protein (MBP), a technique previously shown to enhance allergen production and recognition (Rainyte et al. 2023). This research is the first to investigate rCan f 6 production in the eukaryotic expression system *P. pastoris* and to compare it with rCan f 6 produced in *E. coli*. The comparative analyses were carried out using indirect enzyme-linked immunosorbent assays (ELISA), and the study also examined the impact of cross-reactive carbohydrate determinants (CCD) on the sensitivity of immunochemical tests.

## Materials and methods

### Codon-optimized Can f 6 gene

The *Canis familiaris* Can f 6 gene coding sequence (accession no. HE653774.1) was obtained from GenBank and optimized using the OptimumGene™ Codon Optimization Analysis for efficient gene expression in yeast. GenScript USA Inc. chemically synthesized the codon-optimized sequence (accession no. PQ679325) and supplied it in a pUC57 cloning vector as pUC57/Can f 6.

### Strains and media

Cloning and expression vectors were amplified in *E. coli* DH5α cells. For expression of Can f 6 in bacteria, *E. coli* BL21 (DE3) (Invitrogen, Carlsbad, CA, USA) was used. The PichiaPink™ expression system (Invitrogen, Carlsbad, CA, USA) containing four mutant *P. pastoris* strains (strains 1–4) was utilized to express Can f 6 in yeast. PichiaPink™ Strain 2, an *ade2* and *pep4* knockout, was selected for further experiments. *P. pastoris* growth and storage YPD medium (Yeast Peptone Dextrose; 1% yeast extract, 2% peptone, and 2% dextrose) with and without agar were used. YPDS (YPD medium with 1 M D-Sorbitol) was used to recover *P. pastoris* cells after transformation. *P. pastoris* transformed cells were grown on PAD agar (Pichia Adenine Dropout media; 13.4% YNB-aa, 1.25% CMS-ADE, 0.005% biotin, and 2% dextrose). For the growth and induction of *P. pastoris* cultures, BGMY and BMMY media (Buffered Glycerol-complex Medium and Buffered Methanol-complex medium; 1% yeast extract, 2% peptone, 100 mM potassium phosphate (pH 6.0), 1.34% YNB-aa, 0.00004% biotin, and 1% glycerol or 0.5% methanol) were used.

### Human plasma

Table 1 shows human plasma for rCan f 6 identification and immunochemical characterization. Samples were tested with ImmunoCAP™ and Phadia UniCap™ assays; sIgE scores below 0.35 kU/L were designated negative, and sIgE ≥ 0.35 kU/L positive. Serum pools were prepared by combining individual samples into one mixture used for experiments. Samples were purchased from PlasmaLab International (Everett, WA, USA) and stored at −20°C before testing.

**Table 1:** Characteristics of human plasma samples used in the assays

	No. of samples	*C. familiaris* sIgE	Can f 6 sIgE	Other allergen sIgE	Serum pool
(i)	19	Positive	Positive	Positive	
(ii)	7	Positive	Negative	Positive	Positive-pool
(iii)	7	Negative	Negative	Negative	Negative-pool

### Cloning, expression, and purification of rCan f 6 from *E. coli*

Standard procedures were adhered to for all DNA manipulations, as outlined by Sambrook and Russel (2001). The necessary enzymes, molecular mass standards, and DNA manipulation kits were sourced from Thermo Fisher Scientific Baltics (Vilnius, Lithuania). Utilizing the pUC57/Can f 6 plasmid with primers pET28F and pET28R (Table S1), the Can f 6 gene was amplified using the Pfu DNA polymerase. Subsequent cloning of the resultant DNA fragment occurred at the *BamH*I and *Xho*I sites within pET28a( +) and pET28a( +)-MBP-TEV vectors, gifts from Zita Balklava & Thomas Wassmer (Addgene plasmid #69,929; http://n2t.net/addgene:69929; RRID:Addgene_69929) (Currinn et al. 2016). Sequencing was used to confirm the presence of genes Can f 6 and MBP-TEV-Can f 6, which code an N-terminal hexahistidine-tag (6xHis).

The constructs pET28a( +)-Can f 6 and pET28a( +)-MBP-TEV-Can f 6 were transformed into *E. coli* BL21 (DE3) cells via heat shock. Growth and recombinant protein native purification with some alternations were done according to the manufacturer’s directions (Qiagen, Hilden, Germany). The cells were induced overnight with 1 mM IPTG (Thermo Fisher Scientific Baltics, Vilnius, Lithuania) at 25 °C and then lysed by sonication (4 min; 15 s disruption, 15 s cooling at 20 kHz, and 60% amplitude). The recombinant proteins were purified according to Qiagen’s The QIA*expressionist*™ handbook 5th Ed. Protocol 12. “Batch purification of 6xHis-tagged proteins from *E. coli* under native conditions”. The recombinant Can f 6 (rCan f 6) and recombinant fusion protein MBP-Can f 6 (rMBP-Can f 6) produced in *E. coli* were assessed for purity using sodium-dodecyl sulfate polyacrylamide gel electrophoresis (SDS-PAGE) and Western blot. Concentration measurement involved the Bradford protein assay, using Pierce™ pre-diluted bovine serum albumin (Pierce Biotechnology, Rockford, IL, USA) as the standard. The proteins were then stored at −20 °C, supplemented with 40% glycerol.

### Cloning and expression of rCan f 6 in *P. pastoris*

Can f 6 gene was amplified by PCR using pUC57/Can f 6 plasmid as a template with primers pPinkF and pPinkR (Table S1). Post-amplification, the gene, which now included a C-terminal 6xHis tag sequence, was integrated into the *Stu*I and *Kpn*I restriction sites of the pPink-αF-HC vector (Invitrogen, Carlsbad, CA, USA). This resulted in a plasmid that allowed the expression of Can f 6, aligned with the *Saccharomyces cerevisiae* α-mating factor pre-sequence and controlled by the methanol-activated alcohol oxidase I (AOX1) promoter. The assembled expression vector, pPink-αF-Can f 6, was confirmed by sequencing.

The fusion protein MBP-Can f 6 was created using PCR with pFX7-6xHis-MBP-TEV-Can f 6 plasmid (*unpublished data*) and primers pPinkMBPF and pPinkMBPR (Table S1). The MBP-TEV-Can f 6 gene with an N-terminal 6xHis tag was cloned into the *Stu*I and *Kpn*I restriction sites of the pPink-αF-HC vector. The plasmid pPink-αF-MBP-TEV-Can f 6 was confirmed by sequencing.

The expression of the recombinant proteins Can f 6 (rCan f 6) and MBP-Can f 6 (rMBP-Can f 6) in *P. pastoris* was done as previously described, with the only alteration of the final concentration of methanol being 1% (Rainyte et al. 2023).

### Purification of rCan f 6 from *P. pastoris* culture medium

The gathered *P. pastoris* media was filtered through a 0.45 µm pore PVDF membrane (Stericup Quick Release-HV sterile vacuum filtration system, Merck KGaA, Darmstadt, Germany) and dialyzed against 50 mM NaH_2_PO_4_, 300 mM NaCl, and 10 mM imidazole, pH 8.0 buffer. Before chromatographic purification, the dialyzed media was again filtered through a 0.45 µm pore PVDF membrane. The purification was performed at 4 °C using an ÄKTApurifier 100 chromatography system equipped with a sample pump P-960 and a fraction collector Frac-920 (GE Healthcare Bio-Sciences AB, Uppsala, Sweden). The clarified media was directed onto a Ni–NTA Superflow column (1 mL, Qiagen, Hilden, Germany), pre-equilibrated with the dialysis buffer at a 1 mL/min flow rate. The column was subsequently rinsed with 10 column volumes (CV) of 50 mM NaH_2_PO_4_, 300 mM NaCl, 20 mM imidazole, and pH 8.0 buffer, and a linear imidazole gradient from 20 to 500 mM was used to elute the target protein in 10 CV.

### Purification of rMBP-Can f 6 from *P. pastoris* culture medium

*P. pastoris* culture media was filtered using a 0.45 µm pore PVDF membrane (Stericup Quick Release-HV sterile vacuum filtration system, Merck KGaA, Darmstadt, Germany). The media was then dialyzed against 50 mM acetic acid-NaOH, pH 4.3 buffer for cation-exchange chromatography using SP Sepharose Fast Flow (GE Healthcare Bio-Sciences AB, Uppsala, Sweden), and against 20 mM Tris–HCl, pH 7.5 (4 °C) buffer for anion-exchange chromatography using Q Sepharose Fast Flow (GE Healthcare Bio-Sciences AB, Uppsala, Sweden). Before chromatographic purification, the dialyzed media was filtered through a 0.45 µm pore size PVDF membrane. The purifications were performed at 4 °C using the ÄKTApurifier 100 system equipped with a sample pump P-960 and a fraction collector Frac-920 (GE Healthcare Bio-Sciences AB, Uppsala, Sweden).

Two milliliters of SP Sepharose Fast Flow was packed into a C 10/10 column (GE Healthcare Bio-Sciences AB, Uppsala, Sweden) and equilibrated with 50 mM acetic acid-NaOH, pH 4.3 buffer at a flow rate of 2 mL/min. All subsequent chromatography steps were performed at 1.5 mL/min. The clarified culture media was loaded onto the SP Sepharose FF column. The column was washed with 7 CV of equilibration buffer, and the target protein was eluted with a linear gradient from 0 to 1 M NaCl in 10 CV.

Five milliliters of Q Sepharose Fast Flow was packed into an XK 16/20 column (GE Healthcare Bio-Sciences AB, Uppsala, Sweden) and equilibrated with 20 mM Tris–HCl, pH 7.5 (4 °C) buffer at a flow rate of 5 mL/min. All subsequent chromatography steps were performed at 3.5 mL/min. The clarified culture media was loaded onto the Q Sepharose FF column. The column was washed with 12 CV of equilibration buffer, and the target protein was eluted with a linear gradient from 0 to 1 M NaCl in 10 CV.

### SDS-PAGE and Western blot assay

Protein samples were heated in reducing sample buffer and separated using 12% and 14% SDS-PAGE gels. The proteins were then stained with Coomassie Brilliant Blue (Sigma-Aldrich Co.) for visualization. A Western blot (WB) assay was conducted following the method described by Špakova et al. 2020. Primary antibodies used were 6xHis Tag Monoclonal Antibody (MAb) (MA121315, Invitrogen, Carlsbad, CA, USA) and an in-house produced murine anti-MBP MAb 19C19 obtained from the Institute of Biotechnology, Vilnius University (Sližienė et al. 2023). The secondary antibodies were goat anti-mouse IgG (H + L) HRP conjugate (Cat. Nr. 1,706,516, Bio-Rad, Hercules, CA, USA).

For WB with human plasma, the membrane was prepared after semi-dry electroblotting, and human plasma samples diluted 50-fold in PBS-T were used as primary antibodies. The secondary antibodies included mouse anti-human IgE Fc-HRP conjugate (B3102E8, SouthernBiotech, Birmingham, Al, USA) at a 1:1000 dilution in PBS-T.

### Glycosylation detection

The glycosylation of the recombinant protein was assessed through a lectin-probed WB analysis, a technique that reveals glycan structures of glycoproteins by leveraging the carbohydrate-binding specificity of the chosen lectin (Sato 2014). Concanavalin A (Con A) lectin was selected for this study due to its primary binding affinity to mannose (Maupin et al. 2012). The process was carried out by modifying the previously referenced WB technique. After the membrane was blocked and washed, it was subjected to an overnight incubation at room temperature with *Canavalia ensiformis* (Jack bean) lectin Concanavalin A peroxidase conjugate (Sigma-Aldrich Co.), diluted to 0.1 µg/mL in PBS-T. The membrane was subsequently washed multiple times with PBS-T, and the chromogenic signal was detected using 1-Step™ Ultra TMB-Blotting solution (Thermo Scientific, Waltham, MA, USA).

### Deglycosylation

The deglycosylation process was performed using Protein Deglycosylation Mix II (New England Biolabs, Ipswich, MA, USA), following the guidelines provided by the manufacturer. For this reaction, 100 µg of the purified protein was combined with water to a final volume of 40 µL. Then, 5 µL of the Deglycosylation Mix Buffer 2 was added, and the mixture was incubated at 75 °C for 10 min to promote protein denaturation, followed by cooling to room temperature. Next, 5 µL of Protein Deglycosylation Mix II was added. After gentle mixing, the reaction was incubated at 25 °C for 30 min before being shifted to 37 °C for an additional hour to ensure complete deglycosylation. The final mixture was analyzed by SDS-PAGE and WB methods.

### Indirect enzyme-linked immunosorbent assay (ELISA)

Multiwell Nunc MaxiSorp™ polystyrene plates (Invitrogen, Carlsbad, CA, USA) were coated with 50 µL per well of either the recombinant proteins or the control protein MBP, expressed and purified from *E. coli* (Sližienė et al. 2022). Antigen solutions were diluted at 5 µg/mL in a coating buffer (50 mM NaHCO_3_, pH 9.5) and incubated overnight at 4°C. After incubation, blocking was performed with 300 µL per well Roti®Block solution (Carl Roth Gmbh & Co. Kg, Karlsruhe, Germany) for 2 h at RT and then rinsed twice with deionized water. Human plasma was diluted tenfold with PBS-T, transferred to the plates at 100 µL/well, and incubated at RT for 2 h with mixing at 400 rpm (Biosan Plate Thermo-Shaker, PST-60HL-4). Following a four-time wash with 50% diluted PBS-T buffer, the plates were treated with 50 µL per well of a 1:1000 dilution (with PBS-T) of mouse anti-human IgE Fc-HRP conjugate (SouthernBiotech, Birmingham, AL, USA) for an hour. After four additional washes with 50% diluted PBS-T buffer and two rinses with water, antigen-specific antibodies were detected by adding 50 µL/well of TMB chromogen solution (Invitrogen, Carlsbad, CA, USA). The reaction was halted with the addition of 3.6% H_2_SO_4_ solution, and the optical density (OD) was read at 450 nm with a reference filter of 620 nm in a microplate reader (Sunrise Tecan, Männedorf, Switzerland).

### Statistical analysis

The triplicate ELISA datasets were normalized across multiple plates to account for inter-plate variability, ensuring that the results are comparable regardless of the plate-to-plate differences. Normalization was done by subtracting the mean OD of blank wells accounting for background OD. Then, each sample’s OD was divided by the OD of its respective control (the OD value of *C. familiaris* allergen extract reaction with the positive pool plasma) on the same plate. That removed plate-to-plate variability by scaling the sample’s values relative to the control of that plate. Once all samples were normalized relative to their plate controls, each normalized sample value was multiplied by the mean control OD (the average of all the plate’s positive controls). This step ensures that all plates are adjusted to the same baseline (the total mean), making the data consistent across plates. The triplicate results were presented as the normalized mean OD ± SD. The cut-off value was determined as the normalized mean OD of the negative controls plus three standard deviations.

For statistical analysis, a repeated-measures ANOVA was performed to assess differences in reactivity between recombinant Can f 6 protein variants. The analysis was conducted in Python (via the PyCharm IDE) using the statsmodels library. Tukey’s Honest Significant Difference (HSD) test was performed for post hoc pairwise comparisons. Additionally, paired *t*-tests (t-Test: Paired Two Sample for Means) were performed in Microsoft Excel version 2202 (Build 16.0.14931.20648) to compare specific proteins tested on the same serum samples. Statistical differences were considered significant at *p* < 0.05.

## Results

### *E. coli*-based expression and purification of rCan f 6 allergen variants

**Fig. 1:**
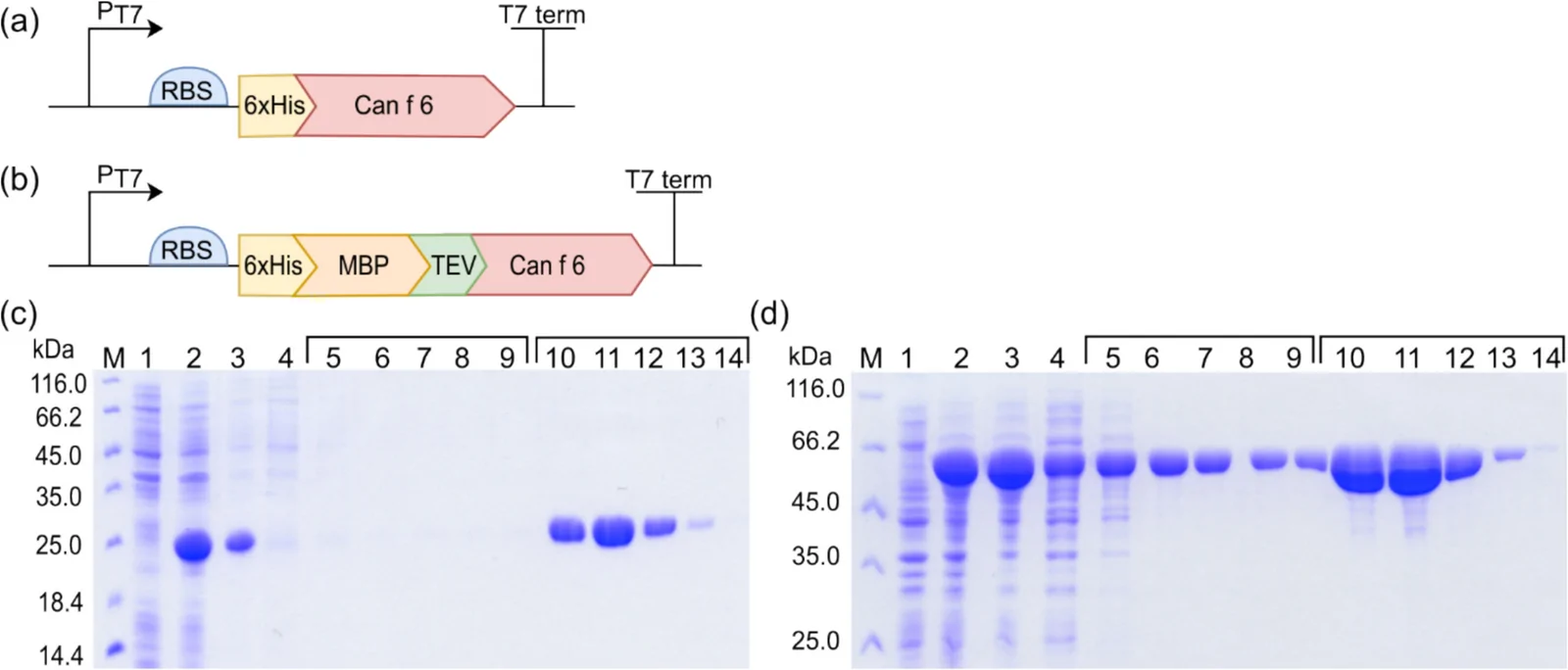
*E. coli*-produced recombinant Can f 6 variant expression and purification. **a, b** Schematic illustrations of gene expression constructs. **a** pET28a( +)-Can f 6 expression vector features the Can f 6 gene tagged at the N-terminus with a 6xHis tag. **b** pET28a( +)-MBP-TEV-Can f 6 expression vector features Can f 6 fused to the C-terminus of a 6xHis-MBP fusion protein, with a TEV protease cleavage site inserted between the fusion partner and the target protein. SDS-PAGE analysis of expression and purification of **c** rCan f 6 and **d** rMBP-Can f 6. Lanes: (1), lysate fraction from non-induced *E. coli*; (2), lysate fraction from IPTG-induced *E. coli*; (3), soluble fraction before purification with nickel-chelate chromatography; (4), soluble fraction after incubation with Ni–NTA agarose; (5–9), wash fractions; (10–14), elution fractions containing purified rCan f 6 and rMBP-Can f 6 proteins, respectively. M—Pierce™ Unstained Protein MW Marker (Thermo Fisher Scientific, USA). Proteins were separated on SDS-PAGE gels of **c** 12% and **d** 14% acrylamide concentrations

In the present study, the 528 bp Can f 6 coding gene was subcloned into two distinct expression systems: (i) pET28-a( +) and (ii) pET28a( +)-MBP-TEV. The goal was to determine if an MBP segment could boost recombinant allergen output (Nallamsetty et al. 2005). In the first vector, an N-terminal 6xHis tag was added to the Can f 6 gene (Fig. 1a). In the second, the Can f 6 was integrated with the N-terminal 6xHis-tagged MBP protein (Fig. 1b). Upon expressing the rCan f 6 constructs in the *E. coli* strain BL21 (DE3), distinct protein bands were observed through SDS-PAGE analysis. Specifically, bands at 23.7 kDa (Fig. 1c) and 64.4 kDa (Fig. 1d) corresponded to rCan f 6 and rMBP-Can f 6, respectively. These were evident in the soluble fractions after IPTG induction (lane 3 in Fig. 1c, d). Before IPTG induction, such bands were notably absent (lane 1 in Fig. 1c, d). Following batch purification of 6xHis-tagged proteins from *E. coli* under native conditions, both rCan f 6 and rMBP-Can f 6 were isolated in their pure forms (lanes 10–14 in Fig. 1c, d). The yields post-nickel-chelate chromatography were 42 mg/L for rCan f 6 and 78 mg/L for rMBP-Can f 6.

### *E. coli*-derived rCan f 6: verification and stability analysis

**Fig. 2:**
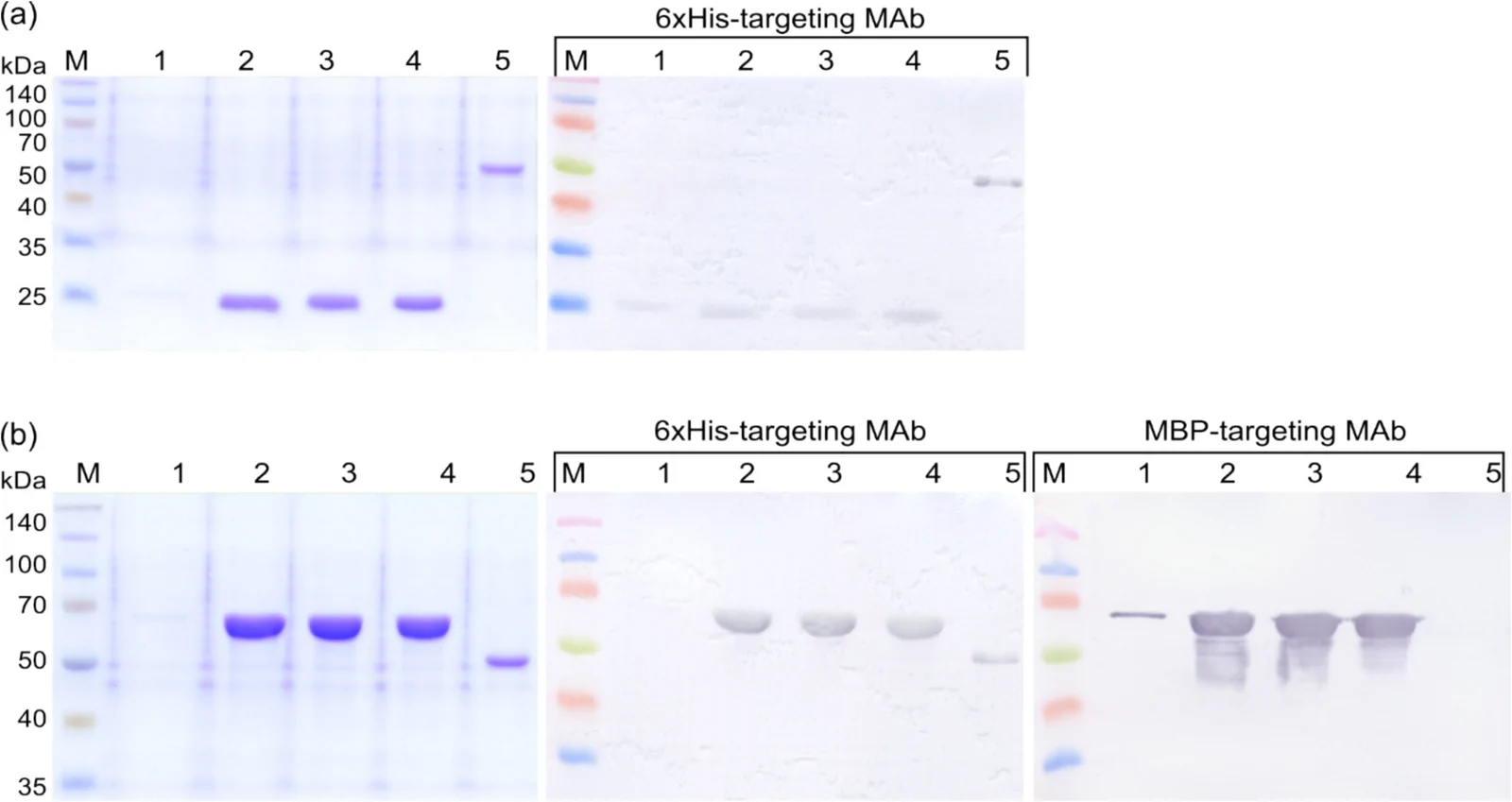
Analysis of purified *E. coli*-produced  **a** rCan f 6 and **b** rMBP-Can f 6 proteins by SDS-PAGE and WB (annotated by the used MAb designation). Lanes: (1), protein samples after lyophilization; (2), protein samples after storage at 4 °C; (3), protein samples after storage at −20 °C without glycerol; (4), protein samples after storage at −20 °C with 40% glycerol; (5), *S. cerevisiae*-produced 6xHis-tagged Hantavirus Sin Nombre nucleocapsid protein (Kucinskaite-Kodze et al. 2011). Lane (5) protein acts as a positive control for WB with 6xHis-targeting MAbs and a negative control for WB with MBP-targeting MAbs. M—Spectra™ Multicolor Broad Range Protein Ladder (Thermo Fisher Scientific, USA). WB analyses were performed using MAbs. Complete WB images, including additional controls, are available in the Supplementary material file (Fig. S1)

The identities of the purified proteins were confirmed using WB analysis with monoclonal antibodies targeting 6xHis and MBP tags (Fig. 2). Additionally, protein stability was assessed after a month of storage under different conditions. As shown in Fig. 2, lyophilization adversely impacts rCan f 6 (Fig. 2a lane 1) and rMBP-Can f 6 (Fig. 2b lane 1). Storage at 4 °C, −20 °C, and −20 °C supplemented with 40% glycerol did not reveal significant changes in the short term (Fig. 2 lanes 2, 3, and 4). However, a follow-up assessment at 24 months (data not presented) indicated marked degradation for samples stored at 4 °C and −20 °C without glycerol. Conversely, samples stored at −20 °C with 40% glycerol exhibited only marginal degradation.

### Expression of rCan f 6 allergen variants in *P. pastoris*

**Fig. 3:**
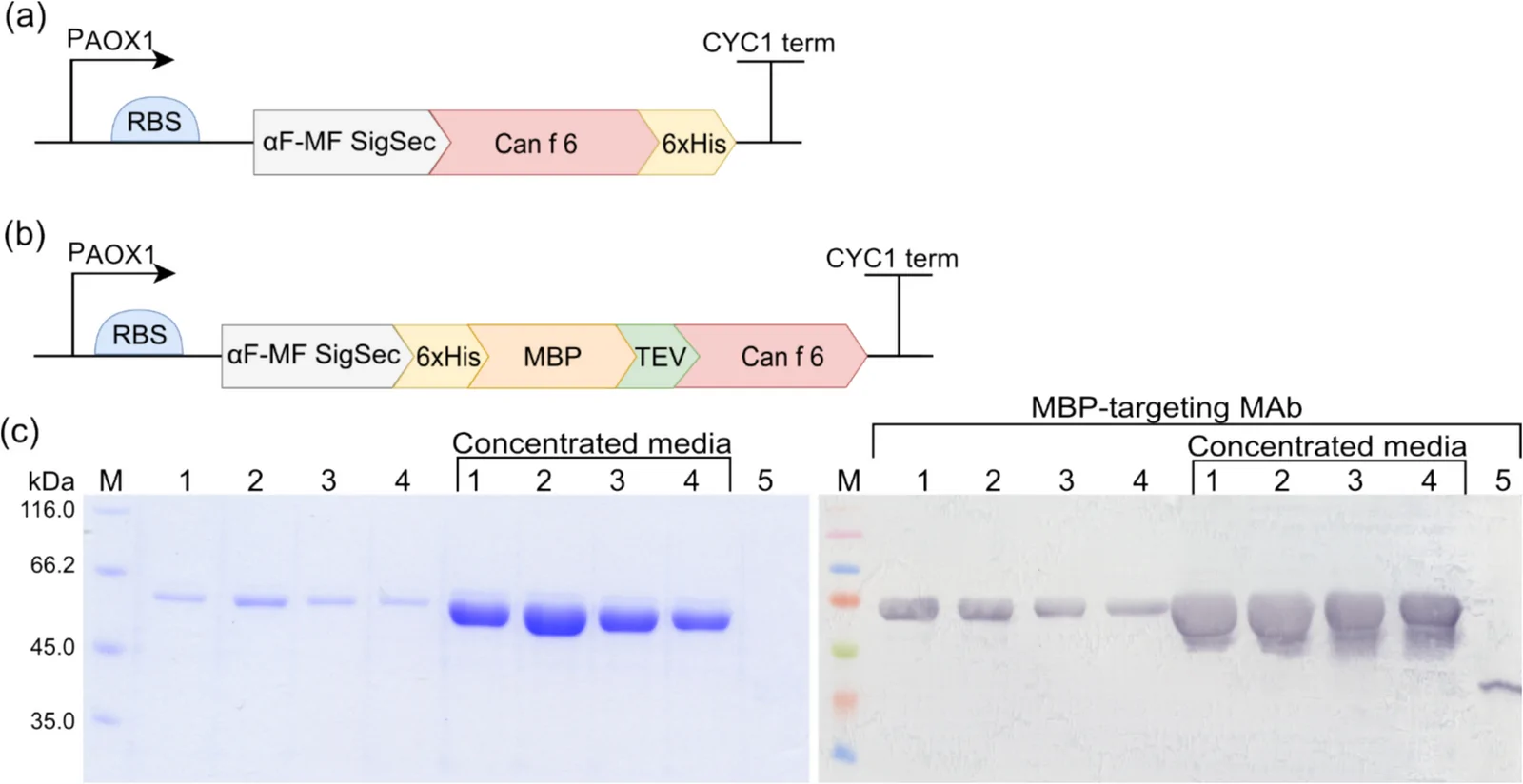
Yeast expression constructs and the comparative analysis of rMBP-Can f 6 expression in *P. pastoris*. **a, b** Schematic representation of gene expression cassettes. In frame with the *S. cerevisiae* alpha-mating factor signal pre-sequence (αF-MF-SigSec) in **a** pPink-αF-Can f 6 expression vector is the Can f 6 gene C-terminally tagged with a 6xHis tag, and in **b** pPink-αF-MBP-TEV-Can f 6 expression vector is Can f 6 fused to the C-terminal end of a 6xHis-MBP fusion protein with a TEV protease recognition site between the fusion and passenger protein. **c** Analysis of media collected after heterologous protein induction in PichiaPink™ yeast strains transformed with pPink-αF-MBP-TEV-Can f 6 expression vector by SDS-PAGE and WB (annotated by the used MAb designation). The lane number corresponds to the particular number of the PichiaPink™ strains. Lane (5) is a positive control—*P.* *pastoris*-produced rMBP. Concentrated media annotates the equivalent media samples, concentrated with methanol 8.7 times. M—Pierce™ Unstained Protein MW Marker and Spectra™ Multicolor Broad Range Protein Ladder (Thermo Fisher Scientific, USA)

**Fig. 4:**
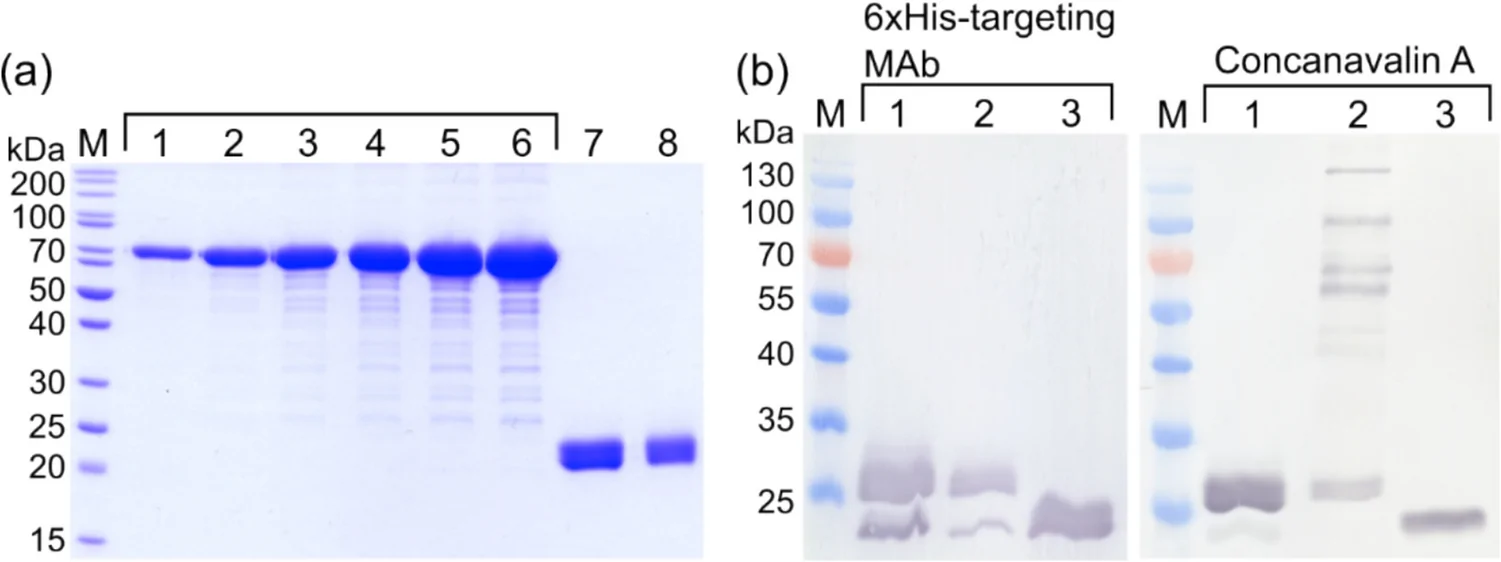
Analysis of the purified *P. pastoris*-produced rCan f 6 protein by **a** SDS-PAGE and **b** WB (annotated by the used MAb designation). In the picture **a**, lanes represent the following: (1–6) correspond to BSA standards with concentrations of 0.25, 0.5, 0.75, 1, 1.5, and 2 mg/mL, respectively; (7) rCan f 6; (8) rCan f 6 diluted by 50%. M—Pierce™ Unstained Protein MW Marker (Thermo Fisher Scientific, USA). In the picture **b**, lanes represent the following: (1) rCan f 6; (2) dialyzed extracellular *P. pastoris* medium containing the secreted rCan f 6; (3) rCan f 6 deglycosylated with PNGase F. M—Spectra™ Multicolor Broad Range Protein Ladder (Thermo Fisher Scientific, USA). Protein samples were fractioned in 14% SDS-PAGE gels. Complete original WB images, including additional controls, are available in the Supplementary material file (Fig. S2)

The PichiaPink™ expression system facilitated the production of rCan f 6 variants in *P. pastoris*. The codon-optimized Can f 6 gene with the 6xHis tag at the C-terminal end of the target gene was subcloned in frame with the *S. cerevisiae* alpha-mating factor pre-sequence in the pPink-αF-HC expression vector, resulting in the expression vector pPink-αF-Can f 6. The rMBP-Can f 6 fusion protein was assembled by subcloning the entire open reading frame of 6xHis-MBP-TEV-Can f 6 fusion protein into the *Stu*I and *Kpn*I restriction sites of pPink-αF-HC expression vector. The pPink-αF-Can f 6 (Fig. 3a) and pPink-αF-MBP-TEV-Can f 6 (Fig. 3b) constructs were verified by sequencing. The PichiaPink™ expression platform consists of four specialized *ade2* yeast strains. These strains are distinct in proteolytic activity: Strain 1 maintains natural protease function, Strain 2 is deficient in *pep4*, Strain 3 lacks *prb1*, and Strain 4 lacks *pep4* and *prb1* activities. Preliminary experiments were conducted to discern which strain would be most adept at producing the desired protein quantity. *P. pastoris*-produced fusion protein rMBP-Can f 6 theoretical MW was about 65 kDa. Following induction with methanol, all strains containing the pPink-αF-MBP-TEV-Can f 6 vector manifested consistent protein bands, as characterized by SDS-PAGE and WB analyses with an MBP-targeting MAb (Fig. 3c). Interestingly, every strain equipped with the pPink-αF-Can f 6 vector exhibited a dual-band pattern upon methanol induction. While the first band aligns with the anticipated 21 kDa weight for rCan f 6, the other seems to represent a glycosylated version of the rCan f 6 protein (Fig. 4b). For scale-up expression, Strain 2 was chosen for both rCan f 6 variants, based on protein quantity and stability.

### Purification and characterization of *P. pastoris*-produced rCan f 6

The dialyzed extracellular *P. pastoris* medium containing the secreted rCan f 6 protein was processed via affinity FPLC using a Ni–NTA Superflow column. The isolated protein purity and quantity were assessed by SDS-PAGE and quantified using ImageJ 1.50b software. The protein is estimated to be completely pure with a yield of 36 mg/L (Fig. 4a lanes 7 and 8). The dual-band pattern seen even after purification was analyzed by a WB with a 6xHis-targeting MAb and a glycosylation assay with ConA (Fig. 4b). Lane 1 (Fig. 4b), which contained the purified rCan f 6, displayed two bands in WB and the glycosylation assay. However, WB depicted both bands with vivid intensity, while the glycosylation assay showed a very intense upper band and a much fainter lower one. This data indicates that both correspond to rCan f 6, with the upper band representing a hyperglycosylated form. Lane 2, containing the extracellular *P. pastoris* medium with the secreted rCan f 6 protein, showed results consistent with lane 1 in the WB with 6xHis-targeting MAbs. However, the glycosylation assay revealed only a prominent upper band and additional higher MW bands, suggesting minimal glycosylation of the lower band, which was mainly observed in concentrated samples. The deglycosylation of rCan f 6 in lane 3 further confirmed these observations.

### Purification and characterization of *P. pastoris*-produced rMBP-Can f 6

**Fig. 5:**
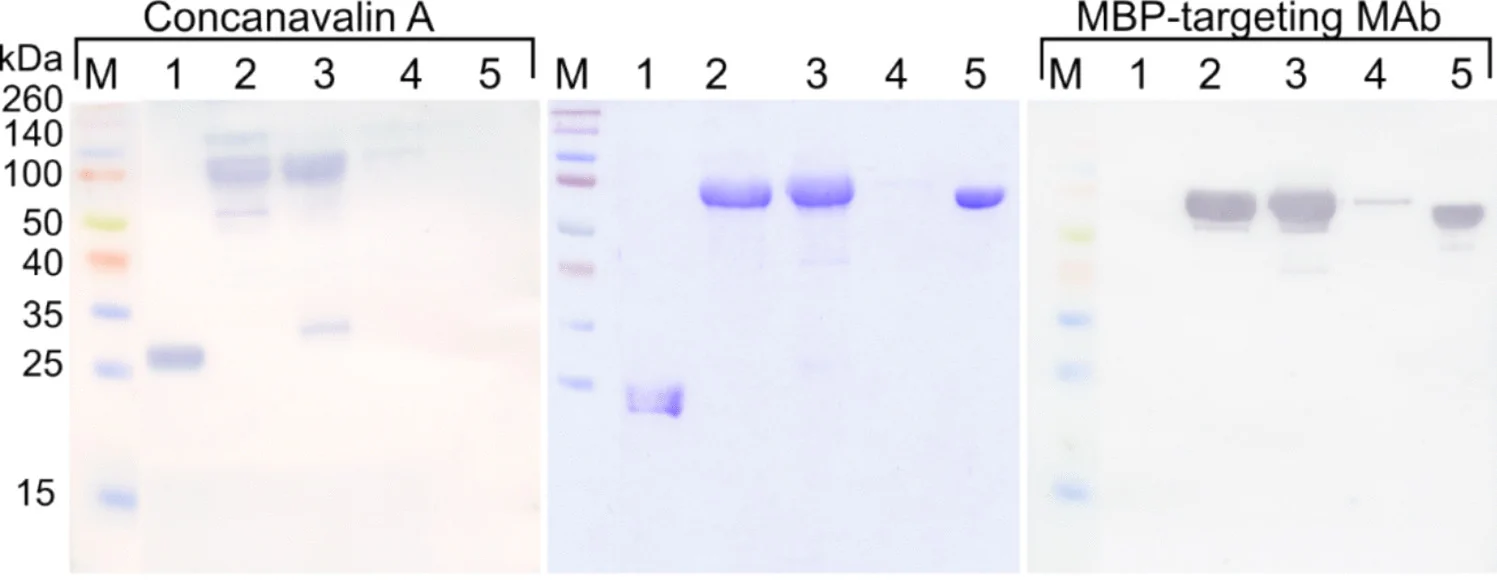
Analysis of purified *P. pastoris*-produced rCan f 6 protein variants by SDS-PAGE and WB (annotated by the used MAb designation). Lanes: (1), rCan f 6; (2), rMBP-Can f 6 purified by AEX; (3), rMBP-Can f 6 purified by CEX; (4), wash fraction from the purification of rMBP-Can f 6 purified by CEX; (5), as an assay control *E. coli*-produced recombinant *Penaeus monodon* allergen rMBP-Pen m 4 was used (Rainyte et al. 2023). M—Spectra™ Multicolor Broad Range Protein Ladder (Thermo Fisher Scientific, USA). Protein samples for WB were fractioned in 14% SDS-PAGE gels. Complete original WB images, including additional controls, are available in the Supplementary material file (Fig. S3)

The rMBP-Can f 6 fusion protein was isolated using two distinct methods: cation exchange (CEX) and anion exchange (AEX) chromatography. The collected fractions, post-purification, were evaluated using SDS-PAGE and the ImageJ 1.50b software. The yields of *P. pastoris*-produced rMBP-Can f 6 purified by CEX and AEX were 18.8 mg/L and 57.5 mg/L, respectively. Further characterization was done using WB with MBP-targeting MAb for protein identification, and Con A lectin was used for glycosylation profiling (Fig. 5). In comparing the results from CEX and AEX, a greater degree of protein degradation was observed in the former. SDS-PAGE gels and WB with MBP-targeting MAb showed multiple bands for the protein sample purified via CEX (Fig. 5 lane 3), suggesting protein fragmentation.

Interestingly, we also note that neither rMBP-Can f 6 protein reacted with MAbs against the 6xHis-tag (data not shown), which suggests that the 6xHis-MBP-TEV-Can f 6 construct is proteolytically cleaved at the N-terminus. Glycosylation analysis revealed comparable glycosylation profiles for both purification methods. However, an additional band corresponding to the MBP tag was observed in the CEX sample (Fig. 5 lane 3), providing further evidence of fragmentation of the fusion protein purified with this method.

### Immunoreactivity of rCan f 6 and rMBP-Can f 6

The three groups of patient plasma were used for testing recombinant Can f 6 proteins (Table 1): (i) 19 individual human plasma samples tested positive for Can f 6 sIgE; (ii) pool-positive, a pool of combined seven human plasma samples, tested positive for *C. familiaris* allergen extract but negative for Can f 6 sIgE; (iii) pool-negative, a pool of combined seven human plasma samples, tested negative for IgE.

**Fig. 6:**
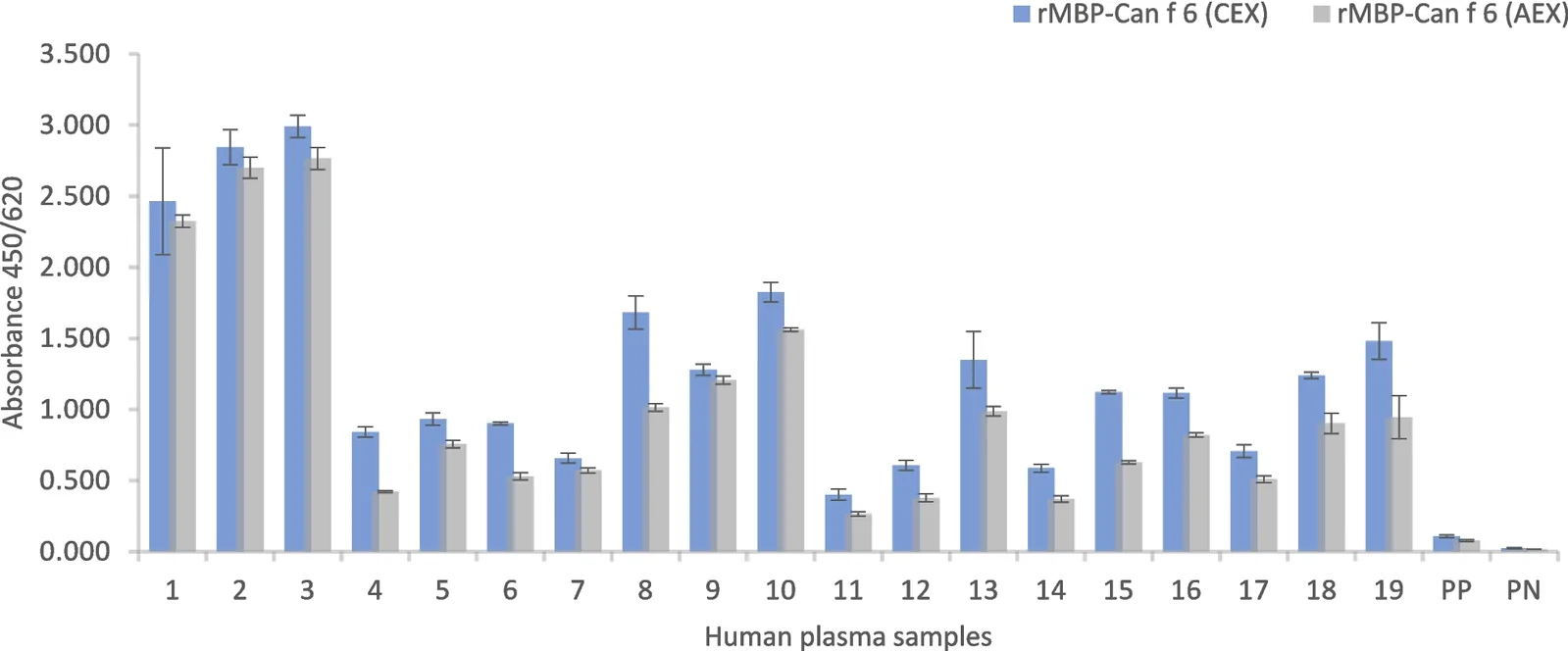
Immunoreactivity analysis of *P. pastoris*-produced rMBP-Can f 6 proteins in an indirect ELISA platform. CEX represents rMBP-Can f 6 purified through cation exchange chromatography, and AEX represents rMBP-Can f 6 purified through anion exchange chromatography. (1–19) correspond to individual plasma from *C. familiaris*-sensitized patients positive for Can f 6; PP—pool-positive (*C. familiaris* sIgE-positive, but Can f 6 sIgE-negative); PN—pool-negative

Firstly, we evaluated the immunoreactivity of rMBP-Can f 6, produced in *P. pastoris*. Indirect ELISA results (Fig. 6) showed that rMBP-Can f 6 purified by CEX reacted significantly stronger than rMBP-Can f 6 purified by AEX to sIgE. A paired student *t*-test with 57 observations (3 replicates per sample) supported the statistical significance with *p* = 1.92 × 10^−16^.

**Fig. 7:**
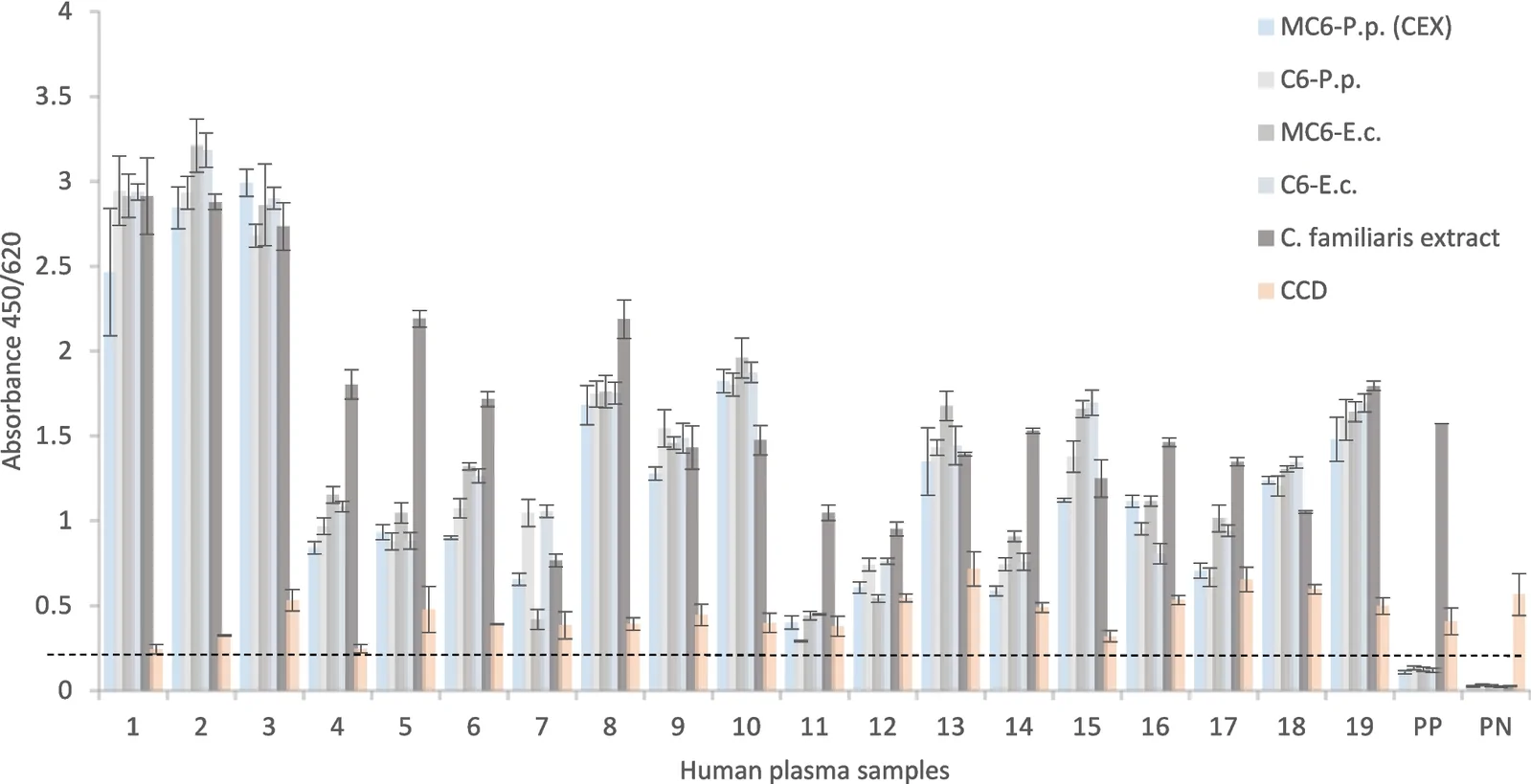
IgE reactivity of patient plasma with recombinant Can f 6 proteins and the *C. familiaris* allergen extract in an indirect ELISA platform. MC6-P.p. (CEX) represents *P. pastoris*-produced rMBP-Can f 6 purified through cation exchange chromatography; C6-P.p. represents *P. pastoris*-produced rCan f 6 protein; MC6-E.c. and C6-E.c. represent *E. coli*-produced rMBP-Can f 6 and rCan f 6 proteins; CCD is the glycosylation IgE marker. (1–19) correspond to individual plasma from *C. familiaris*-sensitized patients positive for Can f 6; PP—pool-positive; PN—pool-negative. A dashed line represents the cut-off

**Fig. 8:**
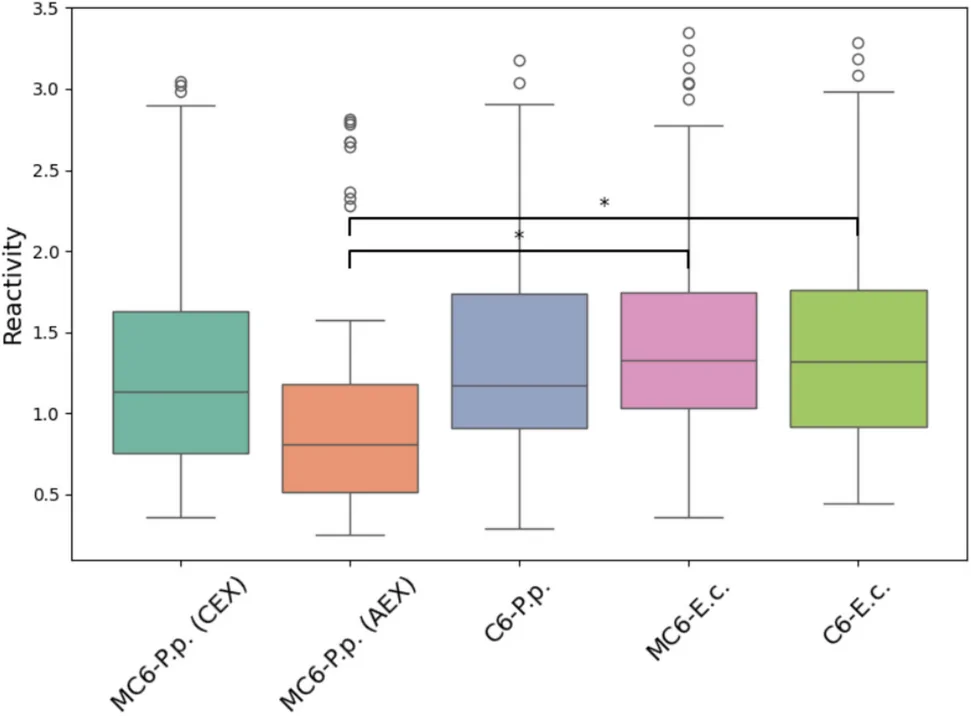
IgE reactivity of patient plasma with recombinant Can f 6 proteins visualized in a boxplot. MC6-P.p. (CEX) represents *P. pastoris*-produced rMBP-Can f 6 purified through cation exchange chromatography; MC6-P.p. (AEX) represents *P. pastoris*-produced rMBP-Can f 6 purified through anion exchange chromatography; C6-P.p. represents *P. pastoris*-produced rCan f 6 protein; MC6-E.c. and C6-E.c. represent *E. coli*-produced rMBP-Can f 6 and rCan f 6 proteins, respectively. The data represents normalized IgE reactivity across 19 individual plasma from *C. familiaris*-sensitized patients positive for Can f 6. Reactivity is represented as normalized OD values, and significant differences between proteins are annotated with asterisks (*p* < 0.05)

Analysis of the absorbance values of all synthesized recombinant Can f 6 proteins (Fig. 7) reveals that all recombinant Can f 6 variants specifically bind to Can f 6 sIgE in human plasma samples with no evidence of non-specific interactions. Repeated measures ANOVA was performed to determine whether recombinant Can f 6 proteins exhibit significant differences in IgE reactivity across serums. Positive and negative control proteins were included in the indirect ELISA to ensure specificity and validate the experimental setup. However, they were excluded from the statistical analysis and comparison of recombinant proteins to avoid artificially inflating variability. The ANOVA analysis revealed significant differences among proteins (*F* (4, 72) = 29.8, *p* < 0.001), indicating variability in reactivity. However, no significant variability was observed between replicates, confirming the consistency of the triplicate measurements. Post hoc comparisons were conducted using Tukey’s HSD test to identify specific protein pairs with significant differences in reactivity. The analysis showed that rMBP-Can f 6 purified through anion exchange chromatography exhibited significantly different reactivity than *E. coli*-produced proteins (Fig. 8). No statistically significant differences were observed between the remaining pairs, suggesting comparable reactivity levels across those groups. Glycosylation analysis was performed passively by adding CCD control and observing any spikes of variability. As can be seen in Fig. 7, no significant differences were found.

## Discussion

Historically, allergen extracts derived from natural sources were used to diagnose and treat allergic diseases. However, these extracts exhibit considerable variability, lack standardization, and contain a complex mixture of allergenic and non-allergenic components (Jeong et al. 2011; Zimmer et al. 2021). The understanding that sufficient and consistent presence of major allergens is decisive for the quality and efficacy of both immunotherapy and diagnostic products led to the revolutionized field of allergy diagnostics (Jutel et al. 2012). One of the key objectives of large-scale European research initiatives, e.g., CREATE (Consortium for the Standardization of Allergen Extracts), BSP090 (Biological Standardization Programme), and MeDALL (Mechanisms of the Developmental Allergy), was the production of high-quality, standardized allergen extracts/molecules with robust reproducibility. While this endeavor was achieved with varying degrees of success, these projects contributed to a better understanding of the molecular mechanisms underlying allergic sensitization and the relationships between sensitization patterns and clinical symptoms while exploring the use of native allergen molecules versus recombinant allergens (Gao et al. 2013; Anto et al. 2017).

Recombinant allergens, produced through genetic engineering, offer a more consistent and precise approach to allergy diagnosis. They facilitate the identification of specific allergens responsible for allergic reactions, a process termed CRD. CRD allows clinicians to distinguish between genuine sensitization and cross-reactivity, refine risk assessment, and provide more personalized therapeutic strategies. Furthermore, recombinant allergens enable the production of hypoallergenic variants, which promise safer and more effective allergen-specific immunotherapy (Chapman et al. 2000; Hiller et al. 2002; Pawankar et al. 2013). As the prevalence of allergic diseases continues to rise globally, the demand for reliable and accurate diagnostic tools becomes ever more pressing. Using recombinant allergens in allergy diagnostics represents a critical step toward achieving more targeted and effective management of allergic disorders (Sánchez-Borges et al. 2018).

Progress in allergy diagnosis and treatment has been notable for allergens related to cats, grasses, and dust mites. However, advancements in addressing dog allergies have lagged. Despite the widespread prevalence of dog allergies, research outcomes on their diagnosis and treatment remain varied and challenging to standardize (Chan and Leung 2018). Research indicates that the allergen content in commercially available dog allergen extracts can be highly inconsistent, with some, like Can f 6, ranging from 3.3% to non-existent. This inconsistency is especially troubling for Can f 6, which is already one of the least prevalent allergens in these extracts. In comparison, Can f 1 allergen content averages 8.7% and Can f 3 at 47.3%, while Can f 6 stands at 1.3%, highlighting its marked scarcity (Wintersand et al. 2019). Monosensitization to Can f 6 remains an under-explored area in allergy research, with a singular reported case highlighting its significance. This particular instance underscores the pivotal role of CRD and molecular-based methodologies. Previous studies indicate that multi-molecular sensitization in dog and horse allergies is associated with increased severity of rhinitis and asthma. Thus, molecular diagnosis serves not just as an allergy marker but can also aid clinicians in forecasting the nature and intensity of clinical symptoms (Uriarte and Sastre 2016; Wintersand et al. 2019).

In this investigation, a lipocalin from *C. familiaris*, known as the allergen Can f 6, was selected to assess the impact of varying expression systems on the antigenic properties of recombinant allergens. The clinical significance of Can f 6 is underlined by its demonstrated ability to trigger specific sensitization (Wintersand et al. 2019) and its established cross-reactivity with cat (67%) and horse-derived (55%) allergens (Nilsson et al. 2012). Several studies have examined recombinant Can f 6 protein expression in *E. coli*; however, no attempts have been made to investigate Can f 6 expression in eukaryotic expression systems (Nilsson et al. 2012; Wang et al. 2017; Yamamoto et al. 2019). This limitation seems like an oversight, as allergen Can f 6, based on sequence analysis, is predicted to have one O-glycosylation site and three N-glycosylation sites while also containing another 10 possible glycosylation sites by random forest algorithm and pairwise pattern prediction tool (Hamby and Hirst 2008). Research on the crystal structure of Can f 6 indicates that the binding between IgE and Can f 6 is highly dependent on its structure (Yamamoto et al. 2019). This finding aligns with other studies demonstrating that lipocalin allergens possess epitopes in their primary and tertiary structures (Hilger et al. 2012).

Protein glycosylation can be ambiguous in allergy diagnostics. On the one hand, glycosylation can be critical for the folding and activity of the recombinant allergen (Bonnet et al. 2018). On the other hand, glycosylation can illicit false positive reactions by reacting with anti-CCD IgE (Kozlov et al. 2025). As allergens can be derived from many different sources, it is common to exhibit native glycosylation patterns. Such carbohydrates are termed “classical CCDs”. They are characterized by the inclusion of non-human monosaccharides (e.g., xylose, fucose) and aberrant glycosylation patterns (e.g., fucosyl residue linked α-(1,3) to the first N-acetyl glucosamine in the N-glycan core) (Platts‐Mills et al. 2021; Potapova et al. 2024). The clinical significance of CCDs is a debatable topic; most believe that classical CCDs do not induce IgE-mediated clinical symptoms as opposed to nonclassical CCDs such as α-Galactose, which was documented to cause an anaphylactic reaction (Homann et al. 2017). However, as this topic is severely understudied, we cannot elucidate CCDs' role in AIT and whether they are of low/non-clinical importance. Anti-CCD IgE are the most disruptive in insect venom allergy diagnostics, as there are many cases of double positivity to honeybee and yellow jacket venom due to anti-CCD IgE binding (Hemmer et al. 2001).

Glycosylation patterns that do not include classical CCD markers, such as mannosylation seen in yeast, are unclear in their interaction with anti-CCD IgE. In most cases, mannosylation does not significantly affect sIgE binding; however, there are a few documented cases where it elicited false-positive results in a studied cohort (Rainyte et al. 2023; Kozlov et al. 2025). Such cases are seen in patients with high anti-CCD IgE antibody levels. Still, such patients can also have false-positive results even with ImmunoCAP assays, where anti-CCD IgE react with the ImmunoCAP cellulose matrix. All these cases can be negated by CCD blockers used before in vitro IgE assays (Hemmer et al. 2018; Kozlov et al. 2025). Alternatively, mutating N-glycosylation sites before expression in yeast can also be an option for in vitro diagnosis. However, for AIT, *P. pastoris* glycosylation could still be useful as mannosylation is the glycosylation pattern for most common allergens, and it lets us produce a recombinant allergen closely related to its native form (Al-Ghouleh et al. 2012).

The selection of the *P. pastoris* expression system for this study was influenced by its inherent advantages. Notably, *P. pastoris* allows high-level expression of proteins, often achieving higher yields than other microbial expression systems. Its ability to perform post-translational modifications (PTM), such as glycosylation, makes it suitable for producing complex mammalian proteins. The easily scalable approach and the lack of endotoxins in its output make *P. pastoris* an effective platform for recombinant allergen production (Schmidt and Hoffman 2002).

As recombinant allergens are designed for clinical use, it must be ensured that specific requirements of purity, stability, safety, and other factors such as consistency, PTMs, and immunogenicity are met. Based on this premise, Can f 6 was fused to the maltose-binding protein. MBP is an established fusion partner in protein expression, particularly in systems like *E. coli*, where it promotes solubility and assists in protein folding (Nallamsetty and Waugh 2006). In theory, MBP also facilitates purification due to its affinity; however, in practice, MBP-fusion proteins often fail to bind to the amylose resin (Nallamsetty et al. 2005; Bell et al. 2013). To circumvent this obstacle, a dual 6xHis-MBP tag was used; this proved to be a correct choice as both rMBP-Can f 6, produced in *E. coli* and *P. pastoris*, failed to bind to the amylose resin (data not shown). These results match earlier studies’ observations (Rainyte et al. 2023). In the context of *P. pastoris*, the efficacy of MBP as a fusion tag remains inconsistent. While it enhances the expression of specific proteins, it does not do so universally, and the underlying reasons for these disparities are not clearly understood (Li et al. 2010).

Consistent with the published literature, rCan f 6 expressed in *E. coli* was soluble. Notably, in the absence of MBP fusion, our production yielded 42 mg/L, surpassing previous results of 21 mg/L achieved with the pGEX4T-2 plasmid (Yamamoto et al. 2019) and 25 mg/L using the pET20b vector (Nilsson et al. 2012). All studies, including ours, employed the *E. coli* BL21 (DE3) strain. When rCan f 6 was co-expressed with MBP, the yield reached 78 mg/L, positioning this method as the most efficient. As the potential clinical applications of rCan f 6 come into focus, shelf-life and stability are paramount. Our data indicate that rCan f 6 undergoes degradation upon lyophilization, irrespective of its MBP fusion. Similar degradation can also be seen during prolonged storage at −20℃ without stabilizing agents like glycerol (data not shown). Given that many commercially available dog allergen extracts are provided in a lyophilized format, the susceptibility of Can f 6 to degradation during the lyophilization process may account for its variability in these extracts (Wintersand et al. 2019). This sensitivity underscores crucial significance when developing products for clinical use.

Considering the inherent limitations of *E. coli*, particularly concerning PTMs and misfolding, we turned to alternative expression systems (Schmidt and Hoffman 2002). In this context, we pioneered the production of rCan f 6 in *P. pastoris*. The PichiaPink™ system is a specialized expression platform based on the yeast *P. pastoris*. It offers streamlined and efficient protein production by incorporating easy strain selection and advanced fermentation protocols, allowing intracellular and secreted protein expression with various selectable markers. rCan f 6 and rMBP-Can f 6 were successfully expressed in yeast. rCan f 6 exhibited a dual-band pattern in SDS-PAGE gels, and further experiments attributed them to higher and lower glycosylation levels. *P. pastoris* glycosylation pattern is formed mainly from mannose residues; this can also be seen in WB with the lectin ConA, which primarily favors mannan. Prior studies have noted the importance of allergen mannosylation, describing it as the dominant glycosylation pattern in most environmental allergens (Al-Ghouleh et al. 2012).

In *P. pastoris*, the production of rMBP-Can f 6 was successful, but the yield notably varied depending on the purification method used. Specifically, cation exchange chromatography yielded 18.8 mg/L, while anion exchange chromatography produced a substantially higher 57.5 mg/L. Despite these differences in yield, the glycosylation patterns between batches remained broadly consistent. However, a notable discrepancy was observed in the degree of degradation, with the cation-purified rMBP-Can f 6 exhibiting more degradation than its counterpart. Interestingly, the main degradation products are proteins with MW around 40 kDa and 25 kDa. These sizes indicate that we are observing proteolyzed fragments of the fusion protein, specifically the separate MBP and Can f 6 proteins. Previous research has pointed to an MBP 3D structure-associated protease, which targets various sites within the region following the MBP domain, encompassing both the spacer and cargo areas (Li et al. 2010). Further experimentation revealed that not just the spacer region but also the cargo region was impacted. Both rMBP-Can f 6 proteins exhibited slight proteolysis from their N-terminal end, which was evident as WB using 6xHis-targeting MAb failed to interact with the rMBP-Can f 6 fusion proteins derived from *P. pastoris*. This observation aligns with our previous research on shrimp allergen Pen m 4 (Rainyte et al. 2023). Despite the observed variances in the two rMBP-Can f 6 proteins, their capability to bind with Can f 6-specific IgE remains unaffected. Both proteins exhibit comparable binding abilities.

The co-expression of rCan f 6 protein with MBP in both systems leads to a heightened yield, affirming it as the most effective production method for rCan f 6 across both expression platforms. Specifically, MBP enhances production by 1.8-fold in *E. coli* and threefold in *P. pastoris*. MBP has successfully served as an expression enhancer for allergens such as Api m 3, Ves v 3, Hev b 1, Phl p 11, and Pen m 4, with no observed detriments to the fusion protein's binding capability (Rihs et al. 2000; Blank et al. 2010; Rainyte et al. 2023; Silimavicius et al. 2024). Our study results further support previous research that MBP is an effective partner for expressing recombinant allergens. Additionally, the fact that companies such as Biomay AG market research-grade recombinant allergens fused with MBP (e.g., Hev b 1, Hev b 5, Hev b 6, and Hev b 11) supports the notion that MBP usually does not inhibit allergenicity. However, it is important to recognize that smaller-sized allergens could be spatially obstructed from interacting with sIgE, as with the peanut allergen Ara h 2 (Mueller et al. 2011). That is why comparing and analyzing recombinant allergens with and without fusion partners is essential.

Antigenicity tests with recombinant Can f 6 proteins revealed notable findings, particularly highlighting significant differences in the reactivity of *P. pastoris*-produced rMBP-Can f 6 proteins. The analysis of indirect ELISA results showed that rMBP-Can f 6 purified by CEX reacted significantly more strongly (*p* < 0.001) to Can f 6 sIgE than rMBP-Can f 6 purified by AEX. We can only speculate that this could be due to the degradation of the cation-purified rMBP-Can f 6, which exposes specific sequential epitopes and makes it more reactive (Wang et al. 2017), or because in CEX, the protein must be positively charged. This positive charge could influence its conformation and stability differently than the negative charge in AEX. Ion exchange chromatography is the gold standard for characterizing therapeutic proteins’ charge variants, so the separation process could inadvertently favor proteins with exposed or intact IgE-binding epitopes (Fekete et al. 2015).

The complete analysis of all the synthesized prokaryotic and eukaryotic proteins showed that all the chosen methodologies are suitable for producing recombinant Can f 6 allergens, as they specifically reacted to Can f 6 sIgE with no non-specific interactions. This analysis confirms that the recombinant proteins retain the essential epitopes required for IgE recognition, validating their suitability for further immunological studies and potential diagnostic applications. However, since basophil degranulation tests were not performed in this study, further investigation is needed to evaluate the allergenic activity of these recombinant allergens for in vivo practices. Additionally, apart from the anion exchange chromatography, purified rMBP-Can f 6 showed no statistically significant differences among the remaining recombinant protein pairs. Glycosylation, often a source of cross-reactivity in in vitro allergy diagnostics, does not influence the IgE reactivity of these recombinant Can f 6 proteins, which eliminates the need for a eukaryotic expression system, as Can f 6 allergens can be effectively produced in *E. coli*.

This study sought to determine whether different expression systems would impact recombinant Can f 6 IgE-binding properties. The results show that *E. coli* and *P. pastoris* systems are equally suitable for rCan f 6 production. N-glycosylation is not critical for folding or recognition of the Can f 6 allergen by sIgE antibodies. Fusion with MBP is a successful strategy for recombinant allergen synthesis and is suitable for downstream applications as it does not react with IgE antibodies. Considering both protein yield and reactivity to sIgE, the most favorable candidate for large-scale production is *E. coli*-produced rMBP-Can f 6 protein.

## Supplementary Information

Below is the link to the electronic supplementary material.

Supplementary file1 (PDF 334 KB)

## Data Availability

The authors confirm that the data supporting the findings of this study are available within the manuscript.
