# Supplementary material for: Enhancing production and assessing IgE reactivity of dog allergen Can f 6 in Pichia pastoris and Escherichia coli
Source: Appl Microbiol Biotechnol. 2025 Mar 29;109:78. doi: 10.1007/s00253-025-13465-7 (PMC11954706; doi:10.1007/s00253-025-13465-7)
Supplement: Supplementary file 1 [file 253_2025_13465_MOESM1_ESM.pdf]

Supplementary information

Enhancing production and assessing IgE reactivity of dog allergen Can f 6 in *Pichia pastoris* and *Escherichia coli*

Juta Dvareckienė[1] • Gintautas Žvirblis[1] • Mindaugas Zaveckas[1] • Rasa Petraitytė-Burneikienė[1]
[1] Vilnius University Life Sciences Center Institute of Biotechnology, Sauletekio av. 7, 10257 Vilnius, Lithuania
✉ Juta Dvareckienė
Corresponding author
juta.rainyte@bti.vu.lt
jutarainyte@gmail.com
Orcid ID: 0000-0003-1910-1509

Gintautas Žvirblis
zvirblis.gintas@gmail.com
Orcid ID: 0000-0001-8753-941X

Mindaugas Zaveckas
mindaugas.zaveckas@bti.vu.lt

Rasa Petraitytė-Burneikienė
rasa.burneikiene@bti.vu.lt
Orcid ID: 0000-0001-5676-2993

Methods and Materials

Table S1. List of primers used in this study.

Primer name	Sequence (5' → 3')	Restriction sites	Tag
pET28F	ATG**GGATCC**CACGAAGAAGAAAACGACGTTG	*Bam*HI	
pET28R	CTT**CTCGAG**TTATTCAGCAGAAGAAACTTGAGC	*Xho*I	
pPinkF	TAC**GAGTCT**AGTCCACGAAGAAGAAAACGACGT	*Mly*I	
pPinkR	TAC**GGTACC**TTAATGGTGATGGTGATGGTGTTCAGCAGAAGAAACTTGAG	*Kpn*I	6xHis
pPinkMBPF	ATG**AGGCCT**ATGGGCAGCAGCCATCATCATCC	*Stu*I	
pPinkMBPR	TAC**GGTACC**TTATTCAGCAGAAGAAACTTGAG	*Kpn*I	

Results

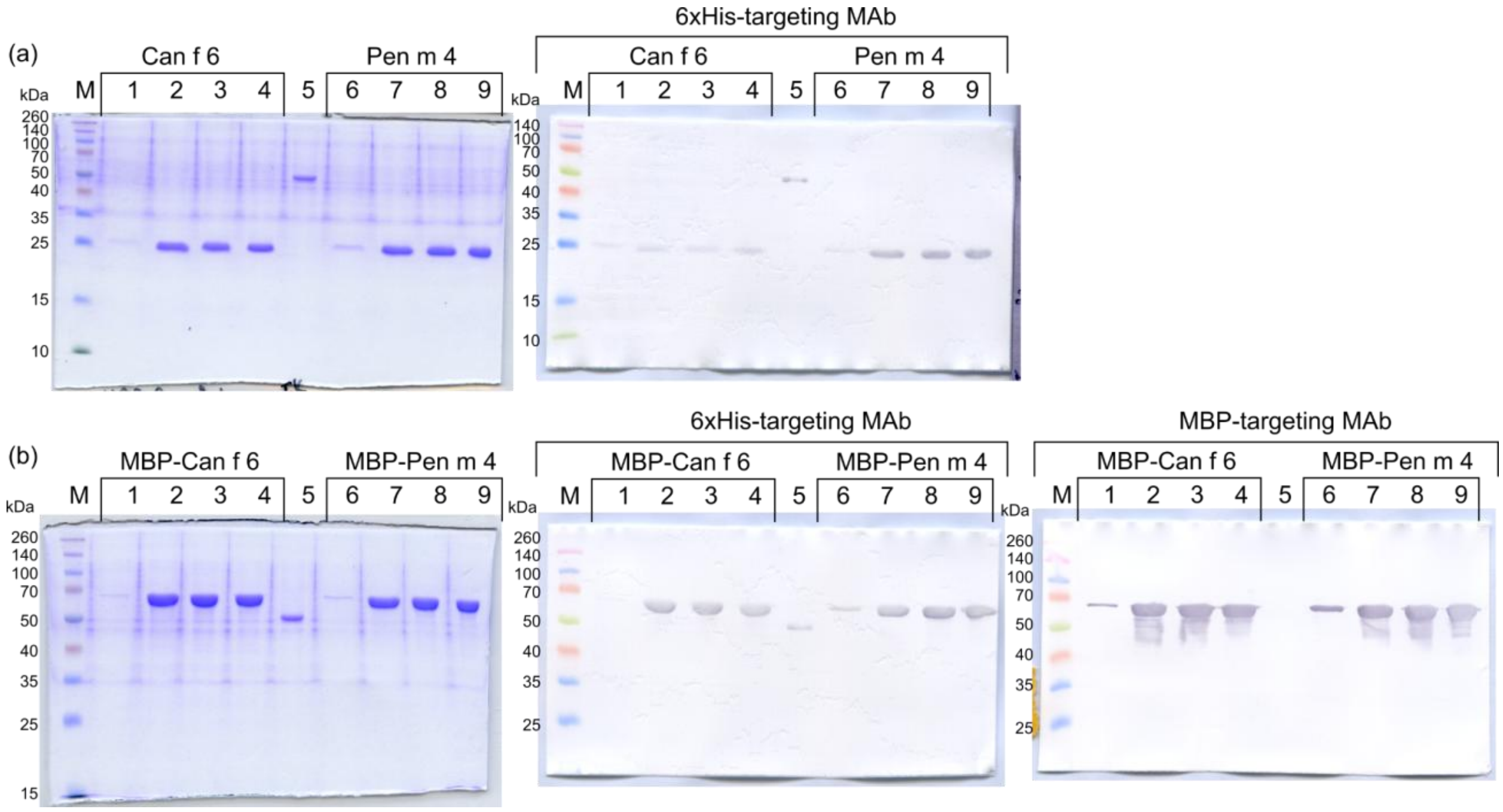

Fig. S1 Original photos of Figure 2. Analysis of purified *E. coli*-produced rCan f 6 and rPen m 4 (a) and rMBP-Can f 6 and rMBP-Pen m 4 (b) proteins by SDS-PAGE and WB (annotated by the used MAb designation). Lanes: [5], *Saccharomyces cerevisiae*-produced 6xHis-tagged Hantavirus Sin Nombre nucleocapsid protein; [1 and 6], protein samples after lyophilization; [2 and 7], protein samples after storage at 4ºC; [3 and 8], protein samples after storage at -20ºC without glycerol; [4 and 9], protein samples after storage at -20ºC with 40% glycerol. Protein samples were analyzed after one month of storage. Lane [5] protein acts as a positive control for WB with MAb against the 6xHis tag and as a negative control for WB with MAb against the MBP tag. M – Spectra™ Multicolor Broad Range Protein Ladder (Thermo Fisher Scientific, USA). WB analyses were performed using MAb.

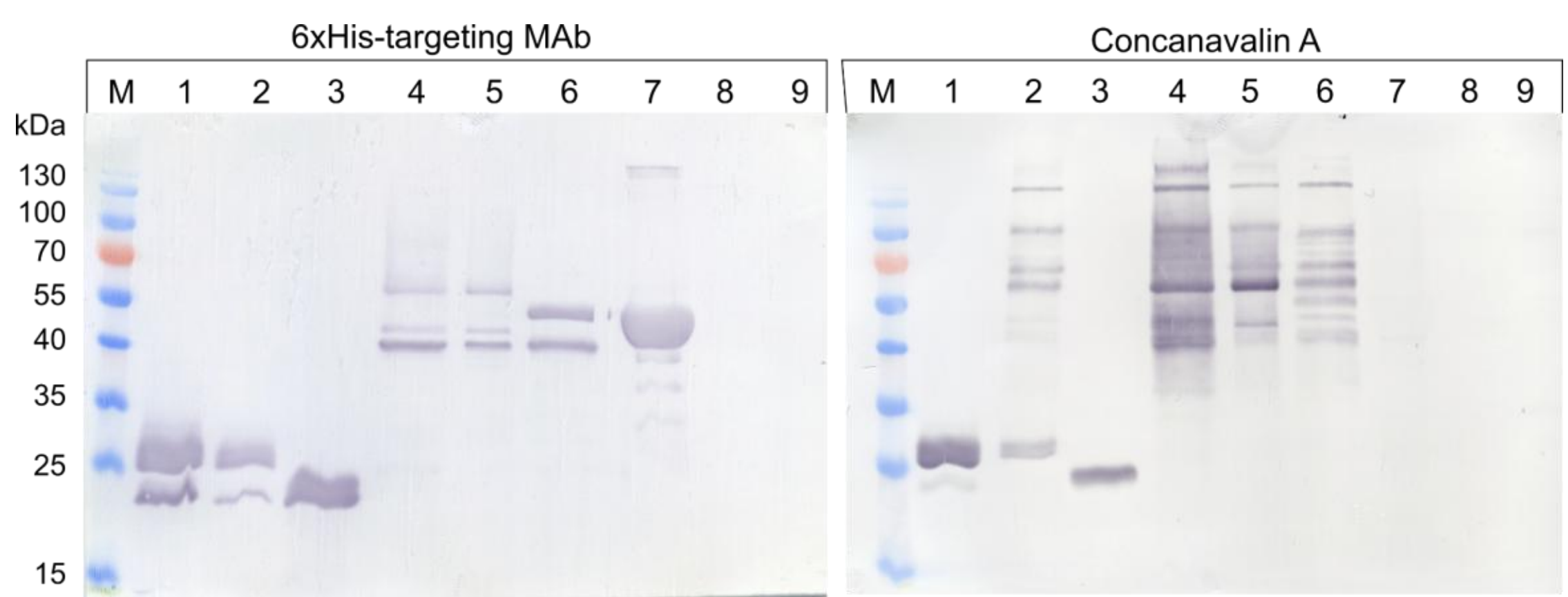

Fig. S2 Original photos of Figure 4. Analysis of purified *P. pastoris*- produced rCan f 6 and rPen m 4 proteins by WB (annotated by the used MAb or lectin designation). Lanes: [1] rCan f 6; [2] dialyzed extracellular *P. pastoris* medium containing the secreted rCan f 6; [3] deglycosylated purified rCan f 6 with PNGase F; [4] dialyzed extracellular *P. pastoris* medium containing the secreted rPen m 4 (concentrated sample); [5] dialyzed extracellular *P. pastoris* medium containing the secreted rPen m 4; [6] dialyzed extracellular *P. pastoris* medium containing the secreted rPen m 4 deglycosylated with PNGase F; [7] *E. coli*-produced rMBP. M – Spectra™ Multicolor Broad Range Protein Ladder (Thermo Fisher Scientific, USA). WB analysis was performed using MAb against the MBP tag. WB glycosylation analysis was performed with ConA lectin. Proteins were separated in 14% SDS-PAGE gels.

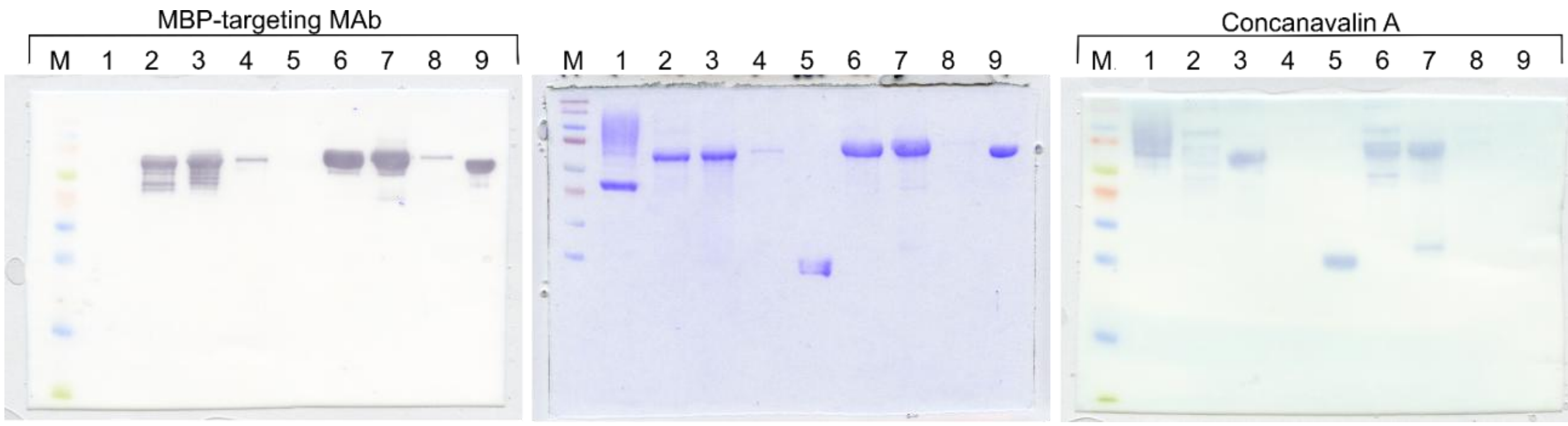

Fig. S3 Original photos of Figure 5. Analysis of purified *P. pastoris*-produced rCan f 6 and rPen m 4 protein variants by SDS-PAGE and WB (annotated by the used MAb designation). Lanes: [1] rPen m 4; [2] rMBP-Pen m 4 purified by anion exchange chromatography; [3] rMBP-Pen m 4 purified by cation exchange chromatography; [4] wash fraction from the purification of rMBP-Pen m 4 purified by cation exchange chromatography; [5] rCan f 6; [6] rMBP-Can f 6 purified by anion exchange chromatography; [7] rMBP-Can f 6 purified by cation exchange chromatography; [8] wash fraction from the purification of rMBP-Can f 6 purified by cation exchange chromatography; [9] *E. coli*-produced recombinant *Penaeus monodon* allergen rMBP-Pen m 4. M – Spectra^{TM} Multicolor Broad Range Protein Ladder (Thermo Fisher Scientific, USA). Protein samples for WB were fractioned in 14% SDS-PAGE gels.
